# A Case of Exogenous Insulin Autoimmune Syndrome: A Case Report

**DOI:** 10.7759/cureus.72067

**Published:** 2024-10-21

**Authors:** Meng Wang, Guangwei Jjiang, Xiangjun Meng, Lina Wang

**Affiliations:** 1 Department of Endocrinology and Metabolism, The First People's Hospital of Linping District, Hangzhou, CHN; 2 Department of Intensive Care Unit, The People's Liberation Army's 903rd Hospital, Hangzhou, CHN

**Keywords:** exogenous insulin autoimmune syndrome, hyperglycemia, hyperinsulinemia, hypoglycemia, insulin autoantibody

## Abstract

Insulin autoimmune syndrome (IAS) is a rare cause of endogenous hyperinsulinemic hypoglycemia triggered by insulin autoantibodies. Through extensive research on IAS in recent years, it has been revealed that the use of exogenous insulin by diabetic patients can result in clinical manifestations similar to those of IAS. This phenomenon is known as exogenous IAS (EIAS). This article describes a case of a patient with EIAS who presented with atypical clinical manifestations. The patient, a middle-aged female with a 17-year history of type 2 diabetes, had been using Insulin Aspart 30 Injection for almost 10 years. She developed severe hyperinsulinemia, low C-peptide levels, positive insulin antibodies, poor postprandial glycemic control, and occasional autonomic nervous system symptoms such as hunger, palpitations, fatigue, and excessive sweating. Despite these symptoms, hypoglycemia was not detected. Switching the type of insulin for two weeks resulted in a significant reduction in insulin dosage, leading to stabilization of fasting and two-hour postprandial blood glucose levels within the target range. This article aims to alert medical professionals about diabetic patients who have hyperinsulinemia, insulin antibodies, and difficulty controlling blood sugar due to EIAS. It is crucial to prevent missed diagnoses, misdiagnoses, and potentially unnecessary surgical interventions through increased awareness.

## Introduction

The first case of the insulin autoimmune syndrome (IAS), also known as Hirata's disease, was reported by Japanese scholar Hirata in 1970 [1], with over 400 cases found worldwide since then. However, there are noticeable racial disparities in its occurrence, as Caucasians rarely experience this syndrome, while it is more prevalent in Southeast Asian countries. Japan has the highest rate, whereas China has a relatively lower rate [2]. China documented its inaugural case of IAS in 1985 [3]. IAS is a rare cause of hypoglycemia characterized by hyperinsulinemia, positive insulin antibodies, and fluctuations between hyperglycemia and hypoglycemia without the use of insulin. Recent research has shown that similar phenomena can also occur in diabetic patients after the administration of exogenous insulin, leading some scholars to refer to it as exogenous IAS (EIAS) [4]. Between 1970 and 2020, research indicates that there have been 120 reported cases of EIAS worldwide, with females accounting for 73 cases and males for 33 cases. The majority of cases occur around the age of 60 [4]. At the same time, studies have shown that 90% of patients with EIAS have type 2 diabetes, while only around 5% have type 1 diabetes. Additionally, the number of EIAS cases is gradually increasing. The condition known as EIAS requires the presence of the following conditions: 1) a history of current or previous use of exogenous insulin; 2) recurring and challenging-to-manage episodes of hyperglycemia, hypoglycemia, or a combination of both; 3) significantly high levels of serum insulin with disproportionately low or reduced C-peptide levels; 4) the presence of positive insulin antibodies; and 5) the exclusion of hypoglycemia caused by other factors [5]. Although the aforementioned diagnostic criteria have not been universally recognized as guidelines or expert consensus, in most cases, both IAS and EIAS are mediated by insulin antibodies and hyperinsulinemia, leading to abnormal glucose metabolism. However, there are also rare cases in which insulin antibodies bind to proinsulin, forming an alternative pathway that results in abnormal glucose metabolism [6]. However, there is a distinction between the two syndromes in terms of the severity of hypoglycemia. Patients with EIAS tend to experience milder hypoglycemic symptoms with a lower frequency of episodes. This may be attributed to the low-capacity, high-affinity nature of the insulin antibodies generated by exogenous insulin, resulting in fewer antigen-antibody complexes being formed, tighter binding, and a reduced likelihood of dissociation [7]. This reported case is an instance of EIAS triggered by the administration of exogenous insulin.

## Case presentation

A 43-year-old female patient was admitted to our hospital in August 2024 after exhibiting consistently high blood glucose levels for 17 years and experiencing uncontrolled blood glucose for the past two months. Seventeen years ago, the patient was found to have increased blood glucose levels during a physical examination, but the specific value was not known. She was diagnosed with type 2 diabetes at a local hospital and was treated with oral hypoglycemic drugs (specific details unknown). After reviewing the medical records, we did not find any information pertaining to the patient from 17 years ago. However, we located the patient's medical records from six years ago, which showed the following: C-peptide (fasting) at 0.81 nmol/L, C-peptide (two-hour postprandial) at 0.94 nmol/L, insulin (fasting) at 257.5 pmol/L, and insulin (two-hour postprandial) at 300.79 pmol/L. The results for the antibodies against glutamic acid decarboxylase, anti-islet cell antibodies, and insulin autoantibodies were all negative. Ten years ago, Aspart Insulin 30 was prescribed for subcutaneous injection before meals due to elevated blood sugar levels. The dosage was set at 20 units for breakfast and 16 units for dinner. She also took voglibose 0.2 mg orally three times a day, metformin hydrochloride tablets 0.5 g orally twice a day, and sitagliptin 100 mg orally once a day to manage her blood sugar levels. Overall, her blood glucose control was deemed satisfactory. The patient had difficulty controlling her blood glucose levels two months ago for unknown reasons. Her self-monitoring of blood glucose (SMBG) reveals that her fasting blood glucose levels fluctuate between 180 and 216 mg/dL, while her postprandial blood glucose readings two hours after meals fluctuate between 234 and 288 mg/dL. She experienced palpitations, hunger, and hand tremors two to three times before meals, which improved after eating. However, she did not monitor her blood glucose levels during this time. To better manage her blood glucose, she sought consultation at our hospital, and the outpatient department diagnosed her with type 2 diabetes, admitting her for further treatment. The patient did not have a past record of hyperthyroidism and reported not using thiol-containing medications like thiamazole, alpha-lipoic acid, and glutathione. Upon physical examination, the following results were observed: temperature of 36.9°C, pulse of 96 beats per minute, respiration rate of 17 breaths per minute, blood pressure of 129/89 mmHg, medium stature, and a body mass index (BMI) of 21.33 kg/m². Decreased temperature sensation, pinprick sensation, and pressure sensation have been noted in both feet. After being admitted, the patient underwent the following additional tests: glycated hemoglobin A1c level was 7.0%, fasting insulin level was greater than 2,152.5 pmol/L, and two-hour postprandial insulin level was also greater than 2,152.5 pmol/L. After dilution, the insulin levels were retested and found to be 9,375.0 pmol/L for fasting and 14,770.0 pmol/L for two-hour postprandial. C-peptide levels were 0.75 nmol/L for fasting and 0.91 nmol/L for two-hour postprandial. Insulin antibodies were measured, and the insulin autoantibody level was greater than 400.0 RU/mL. The antibodies against glutamic acid decarboxylase and anti-islet cell antibodies were all negative. Urine routine test results showed no presence of glucose or ketone bodies. Triglycerides are at 1.07 mmol/L, total cholesterol is at 3.49 mmol/L, and low-density lipoprotein cholesterol is at 2.31 mmol/L. The electrophysiological examination indicates a decreased conduction velocity in the superficial peroneal nerve and sural nerve. A color Doppler ultrasound was performed on the liver, gallbladder, pancreas, spleen, and both kidneys, with no abnormalities noted in the pancreas. An abdominal CT scan also showed no abnormalities in the pancreas. Following admission, the patient was given 20 units of Insulin Aspart 30 Injection subcutaneously before breakfast and 16 units before dinner, along with 0.2 mg of Voglibose orally three times a day, 0.5g of Metformin Hydrochloride Tablets orally twice a day, and 100 mg of Sitagliptin Tablet orally once a day for hypoglycemic treatment. Despite adjustments to the dosage, the patient's blood glucose levels remained unstable, with varying fasting readings between 6.2 and 9.9 mmol/L and two-hour postprandial readings between 3.5 and 16.6 mmol/L. Due to concerns about IAS, Insulin Aspart 30 Injection was discontinued and replaced with Insulin Degludec/Insulin Aspart Injection, leading to a significant improvement in blood glucose levels within two days. The insulin dosage was gradually decreased to prevent hypoglycemia, with the patient being discharged on 15 units before breakfast and 10 units before dinner of Insulin Degludec/Insulin Aspart Injection. The oral medication regimen was also simplified to 0.2 mg of Voglibose three times a day and one tablet of Sitagliptin Metformin (50 mg-0.5 g) twice a day. Following the switch in insulin, the patient no longer experienced symptoms such as palpitations, hand tremors, or hunger. Following discharge, the patient gradually decreased their dose of Insulin Degludec/Insulin Aspart Injection to 12 units before breakfast and eight units before dinner. Their fasting blood glucose remained stable at around 4-5 mmol/L, while two-hour postprandial blood glucose levels ranged between 5 and 8 mmol/L, showing a continued decrease in blood glucose levels. A retest of insulin levels conducted three weeks after switching insulin types showed elevated levels: fasting insulin > 2,152.5 pmol/L, two-hour insulin > 2,152.5 pmol/L. The insulin levels were re-monitored after eight weeks. The fasting insulin level was 8,244.0 pmol/L, and the two-hour postprandial insulin level was 12,650.0 pmol/L. The slight decrease in the patient's insulin levels may be attributed to the brief discontinuation of Insulin Aspart 30. In conclusion, after a decade of Insulin Aspart 30 Injection use, the patient experienced significant increases in fasting and postprandial blood glucose levels. Monitoring of insulin and C-peptide levels revealed elevated insulin autoantibody levels. Switching insulin types led to reduced dosage and stable blood glucose levels within the desired range, clearly indicating a diagnosis of EIAS in this patient.

## Discussion

The IAS is characterized by high levels of insulin, the presence of insulin antibodies, and fluctuations in blood sugar levels, all happening without insulin administration. Many patients with this syndrome have taken medications containing thiol compounds in the past. It is believed that the thiol reacts with insulin's disulfide bond, altering the structure of the body's own insulin and triggering the production of insulin antibodies [8]. Consuming carbohydrates triggers the release of more insulin in the body, causing insulin to form a "reservoir" with antibodies that prevent its hypoglycemic effect, leading to high blood sugar. When insulin separates from these antibodies, hypoglycemia occurs. The external IAS refers to hyperinsulinemia, positive insulin antibodies, hyperglycemia, and hypoglycemia caused by the use of insulin. The occurrence of this situation in EIAS is possibly linked to the quality of insulin injections, the composition of insulin, and the immune status of the patient [9,10]. Research has shown a strong connection between human leukocyte antigens (HLA) and the incidence of IAS, with class II HLA genes playing a confirmed essential role in the onset of autoimmune diseases in humans [11]. Some studies have found that the HLA-DRB1*0406 allele is more associated with IAS in the Asian population following the use of thiol-containing drugs [12]. However, the susceptibility of HLA genotypes to autoimmune hypoglycemia caused by EIAS has not been clearly defined. Patients with EIAS may experience similar immune reactions. Studies have shown that approximately 6.5% of these patients also have concurrent autoimmune diseases, such as Hashimoto's thyroiditis and diffuse toxic goiter [4]. EIAS is characterized by hyperinsulinemia, positive insulin antibodies, hyperglycemia, hypoglycemia, or alternation between the two after the administration of exogenous insulin. Among these, the majority of patients using premixed insulin account for 62% of cases [13]. Studies have shown that the onset of EIAS can occur anywhere from a few days to several decades after insulin use, with a majority of cases manifesting more than ten years later [14]. The symptoms of hyperglycemia and hypoglycemia resulting from EIAS are much less severe compared to those of IAS. This mirrors the clinical presentations of the patients discussed in this article. One possible explanation is that the development of insulin antibodies following exogenous insulin injection leads to the creation of immune complexes through antigen-antibody interaction, preventing insulin from effectively lowering blood sugar levels [15]. However, C-peptide is a metabolite of insulin and does not participate in this process. This leads to hyperinsulinemia and low C-peptide levels, resulting in a dissociation phenomenon between insulin and C-peptide. Studies have shown that the binding of insulin antibodies to exogenous insulin affects insulin reserves, leading to a massive dissociation of insulin that can result in excessive insulin levels in the blood, ultimately causing hypoglycemia. However, due to the different half-lives of C-peptide and insulin, there are no significant fluctuations in C-peptide levels [16]. Studies have indicated that the ratio of insulin to C-peptide can be utilized to predict the presence of EIAS. A fasting insulin/fasting C-peptide ratio greater than 8.6, or a two-hour postprandial insulin/two-hour postprandial C-peptide ratio greater than 17.6, suggests the existence of insulin antibodies in the patient's body, potentially leading to EIAS [17]. Moreover, a higher insulin/C-peptide ratio is correlated with an increased likelihood of developing EIAS and IAS [18,19]. With the advancement of research, the free insulin index (FII), which indicates the proportion of free insulin to total insulin, can be utilized to assess IAS. Patients with IAS exhibit a notable decrease in their FII. A study conducted at Peking Union Medical College Hospital in Beijing, China, revealed that following three days of treatment with hormones in conjunction with mycophenolate mofetil, the FII notably rose to 18.26%, and symptoms of hyperglycemia and hypoglycemia showed significant improvement [20]. The patient described in this article exhibited extremely high insulin levels, so much so that the specific value was undetectable in the initial test. Following sample dilution, the fasting insulin level was determined to be 9,375.0 pmol/L, and the insulin level two hours post-meal was measured at 14,770.00 pmol/L. Surprisingly, the C-peptide level was not elevated but rather low, with a fasting C-peptide reading of 0.75 nmol/L and a post-meal C-peptide level of 0.91 nmol/L. These findings are consistent with previous reports on exogenous insulin-associated autoimmune syndrome at home and abroad. As research on EIAS continues to deepen, it has been discovered that the proportion of elevated insulin levels is relatively low. About 50% of insulin levels are concentrated between 300 and 1,000 uIU/mL [4]. This can also explain why the hyperglycemic and hypoglycemic symptoms in patients with EIAS are relatively milder compared to those with IAS. Studies have also shown that the degree of hyperglycemia and hypoglycemia is related to the intrinsic dissociation rate constant (K-1) and intrinsic affinity [6]. Patients with EIAS typically have relatively low intrinsic dissociation rates and affinities. These factors may explain why the symptoms experienced by these patients are often milder in comparison to those with IAS. As a result, most patients with EIAS have a self-limiting condition and a positive prognosis. In general, symptoms of hyperglycemia and hypoglycemia can be corrected by discontinuing insulin or switching to a different type of insulin within a span of three to six months. However, it may take over a year for insulin antibodies to reach negative levels and for insulin levels to return to normal [19]. Mild EIAS can be improved by eating small meals frequently, reducing the amount of carbohydrates, and increasing protein intake in order to address postprandial hyperglycemia and preprandial hypoglycemia. If the insulin dose is minimal, it may be possible to discontinue insulin and switch to medications such as alpha-glucosidase inhibitors, dipeptidyl peptidase-4 (DPP-4) inhibitors, and glucagon-like peptide-1 receptor agonist (GLP-1RA)to manage blood sugar levels while also preventing hypoglycemia [21,22]. EIAS is an autoimmune disease, so it can be treated with glucocorticoids or immunosuppressants. Therefore, in some refractory cases of EIAS, the use of hormones, immunosuppressants, and plasma exchange may be necessary [23,24]. Some studies have indicated that treatment methods and prognosis do not significantly differ between IAS and EIAS. Nevertheless, IAS patients experience prolonged hypoglycemia symptoms, whereas EIAS patients exhibit less noticeable and shorter-lasting hypoglycemia symptoms [25], similar to those discussed in this article.

## Conclusions

EIAS caused by exogenous insulin is rare and can lead to delays in clinicians' understanding of the disease, resulting in delayed diagnosis and treatment. Patients with EIAS must first discontinue their previous insulin injections. Mild cases of EIAS can be managed by consuming smaller, more frequent meals, reducing carbohydrate intake, and increasing protein intake in order to modify the pattern of postprandial hyperglycemia and preprandial hypoglycemia. If glycemic control remains inadequate, changing the type of insulin or using medications like alpha-glucosidase inhibitors, DPP-4 inhibitors, and GLP-1RA can help lower blood glucose levels while preventing hypoglycemia. In cases of refractory EIAS, corticosteroids, immunosuppressants, and plasma exchange may be necessary.
